# Validation of MRI quantitative susceptibility mapping of superparamagnetic iron oxide nanoparticles for hyperthermia applications in live subjects

**DOI:** 10.1038/s41598-020-58219-9

**Published:** 2020-01-24

**Authors:** Kofi Deh, Marjan Zaman, Yogindra Vedvyas, Zhe Liu, Kelly McCabe Gillen, Padraic O’ Malley, Dina Bedretdinova, Thanh Nguyen, Richard Lee, Pascal Spincemaille, Juyoung Kim, Yi Wang, Moonsoo M. Jin

**Affiliations:** 1 Department of Radiology, Weill Cornell Medicine, New York, NY 10065 USA; 2 Department of Biomedical Engineering, Cornell University, Ithaca, NY 14853 USA; 3 Department of Urology, University of Florida, Gainesville, FL 32610 USA; 4 Urology, Weill Cornell Medicine, New York, NY 10065 USA; 5 Department of Advanced Materials Engineering, Kangwon National University, Samcheok, 245-711 South Korea

**Keywords:** Targeted therapies, Therapeutics, Biomedical engineering

## Abstract

The use of magnetic fluid hyperthermia (MFH) for cancer therapy has shown promise but lacks suitable methods for quantifying exogenous irons such as superparamagnetic iron oxide (SPIO) nanoparticles as a source of heat generation under an alternating magnetic field (AMF). Application of quantitative susceptibility mapping (QSM) technique to prediction of SPIO in preclinical models has been challenging due to a large variation of susceptibility values, chemical shift from tissue fat, and noisier data arising from the higher resolution required to visualize the anatomy of small animals. In this study, we developed a robust QSM for the SPIO ferumoxytol in live mice to examine its potential application in MFH for cancer therapy. We demonstrated that QSM was able to simultaneously detect high level ferumoxytol accumulation in the liver and low level localization near the periphery of tumors. Detection of ferumoxytol distribution in the body by QSM, however, required imaging prior to and post ferumoxytol injection to discriminate exogenous iron susceptibility from other endogenous sources. Intratumoral injection of ferumoxytol combined with AMF produced a ferumoxytol-dose dependent tumor killing. Histology of tumor sections corroborated QSM visualization of ferumoxytol distribution near the tumor periphery, and confirmed the spatial correlation of cell death with ferumoxytol distribution. Due to the dissipation of SPIOs from the injection site, quantitative mapping of SPIO distribution will aid in estimating a change in temperature in tissues, thereby maximizing MFH effects on tumors and minimizing side-effects by avoiding unwanted tissue heating.

## Introduction

Magnetic fluid hyperthermia (MFH) is induced by applying an alternating magnetic field (AMF) to target tissues containing high levels of superparamagnetic iron oxide (SPIO) nanoparticles^1–3^. Hyperthermia therapy is being investigated as a primary or adjuvant therapy for its ability to control and focus cytotoxic effects on target tissues^4–6^. Since the therapeutic effects are proportional to SPIO concentrations in tissue, the ability to accurately predict spatiotemporal distribution of SPIO will be crucial to deliver and monitor optimal therapy^7,8^. Noninvasive methods for quantifying SPIO concentrations *in vivo* include PET imaging of radiolabeled SPIO^9,10^ and various MR imaging techniques including widely used R2* relaxometry using T2*-weighted MRI^11^. R2* measurement was shown to be linear with the concentration of SPIO^12^, and has been applied to various preclinical models of disease^13–15^. However, as R2*-based estimations of SPIO concentration are dependent on imaging parameters as well as the SPIO’s local tissue environment and chemical composition^13,16,17^, it may not be suitable for quantification of an absolute amount of SPIO. Furthermore, R2* measurements saturate rapidly as SPIO approaches the concentration typically used in hyperthermia experiments^18^.

Quantitative susceptibility mapping (QSM) overcomes these R2* problems by exploiting the well defined dipole model of magnetic field generated by magnetic susceptibility sources^19^, particularly by the presence of physiological and pathological iron in tissue^20–24^. QSM extracts magnetic field from the MRI phase data and deconvolutes the field into a magnetic susceptibility distribution using Bayesian inference^19,25^. The concentration of the contrast agent can then be determined by dividing the tissue magnetic susceptibility values in the region of interest (ROI) by the molar susceptibility. *In vitro* experiments have been performed to demonstrate that unlike R2*, the magnetic susceptibility is independent of imaging parameters and the local environment of SPIO^17,26,27^, and is linear within a broad range of concentrations^19^, which makes QSM better suited for absolute quantification of SPIO *in vivo*^28–30^.

The purpose of this work was to demonstrate the feasibility of using QSM to quantify in live subjects a wide range of SPIOs after intravenous or intra-tissue injections intended for hyperthermia effects under AMF^31^. For validation of QSM accuracy for SPIO quantification, we conjugated the clinically approved SPIO ferumoxytol with ^89^Zirconium (^89^Zr) to compare *in vivo* distribution quantitatively by PET and MRI QSM. In addition to validation by imaging-based quantification, tumors were excised and the amount of ferumoxytol within them was quantified by gamma counter. A quantitative correlation between QSM and PET estimation of ferumoxytol concentration was observed, demonstrating the feasibility of using QSM to predict SPIO distribution in tissues. When ferumoxytol was tested for MFH applications in live mice, tumor reduction was found to be specific to the combination of AMF and ferumoxytol. From histological analysis of tumor sections, the region of cell death appeared to correlate with the distribution of ferumoxytol.

## Results

**Figure 1:**
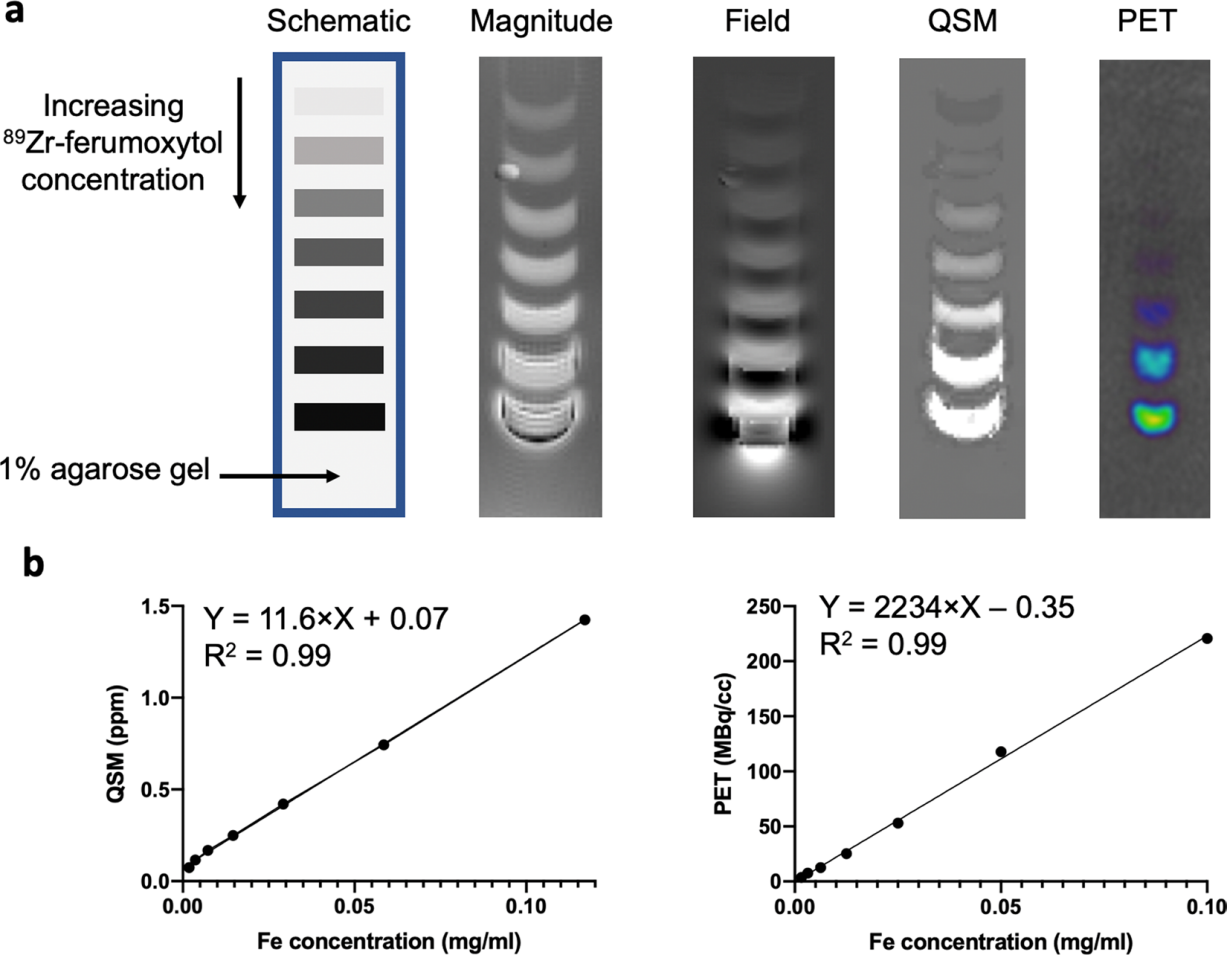
Construction of ^89^Zr-labeled imaging phantom to determine detection sensitivity and conversion factors. (**a**) Images of a representative phantom with MRI and PET images. The phantom consists of a serial dilution of ^89^Zr-ferumoxytol suspended in 1% agarose in PBS solution successively layered on beds of 1% agarose gel in a falcon tube. The concentration of ferumoxytol, estimated from absorbance at 370 nm, was 117, 58.5, 29.3, 14.6, 7.3, 3.7, 1.8 μg/mL by mass of Fe in 200 μl volume. The first echo of the magnitude image, the local field, and QSM images are shown. Note that a rectangular mask was applied to the field to eliminate the extremities of the falcon tube prior to QSM reconstruction. The tube was placed parallel to the main magnetic field in a 7T MR scanner. PET images of the same falcon tube were acquired immediately after the MRI scan. (**b**) Linear regression of ^89^Zr-ferumoxytol phantoms measured by PET and QSM versus the known concentration of ferumoxytol. The slope of the liner curve was used to convert PET and QSM signals to ferumoxytol concentration in live mice and in harvested tissues.

We first examined the accuracy of QSM for estimating ferumoxytol nanoparticle concentrations *in vitro*. In imaging phantoms containing ^89^Zr-ferumoxytol, MR QSM and PET were sensitive enough to detect as low as 1.8 μg Fe/mL, and their measurement increased linearly with ferumoxytol concentration (Fig. 1). QSM linearly responded to ferumoxytol at higher concentrations where the quality of the magnitude image began to decline (Fig. 1a). The conversion factors to relate QSM (ppm) and PET (MBq/cc) units to the concentration of ferumoxytol were derived from linear regression of phantom imaging data (Fig. 1b). The empirical conversion factor to relate QSM to ferumoxytol concentration was determined to be 11.6 ppm×L/g. This conversion factor closely agreed with a theoretical mass susceptibility value of 12.7ppm×L/g at 7 T MRI, which can be derived from the equation relating the mass susceptibility (*χ*_*Fe*_) to mass magnetization (*M*_*Fe*_)^21,32^,

$${\chi }_{Fe}(\frac{{m}^{3}}{kg})=\frac{{\mu }_{0}(\frac{G}{Oe}){M}_{Fe}({B}_{0})(emu/g)}{{B}_{0}(G)}\times 4\times {10}^{-3}\frac{{m}^{3}k{g}^{-1}}{emu\cdot {g}^{-1}\cdot O{e}^{-1}}$$

where *μ*_0_ is the permeability of free space and *B*_0_ is the applied field.

**Figure 2:**
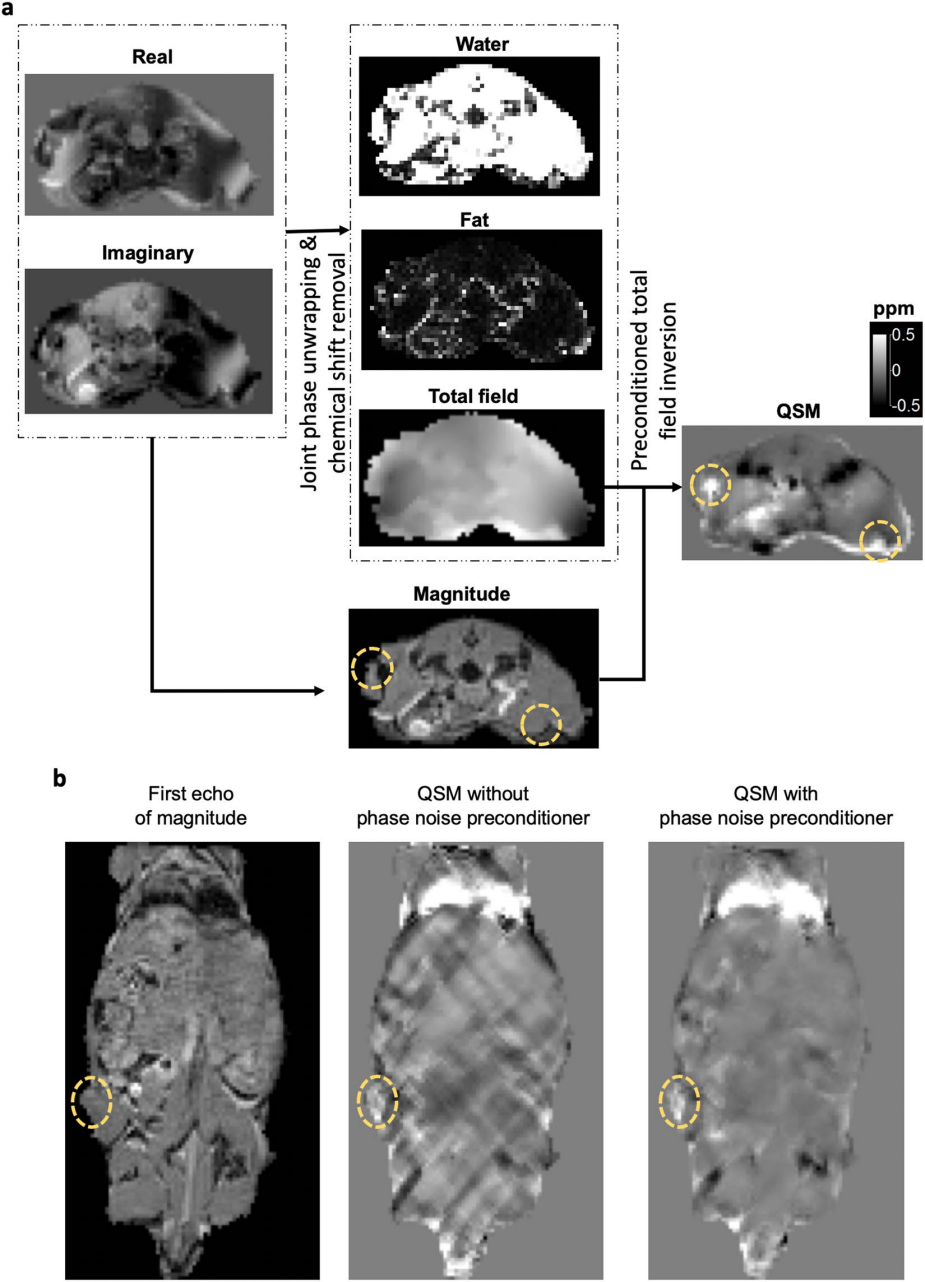
MR QSM and application to detect SPIO in live subjects. (**a**) MR post-processing steps to generate QSM from GRE complex DICOM images. Yellow dotted circles are drawn on the magnitude map to indicate the location of SPIO injections. (**b**) Intravenously injected ferumoxytol was identified by QSM. The yellow dotted circle on the magnitude image points to subcutaneous PC3 tumor.

In comparison with the straightforward process of determining ferumoxytol by QSM in the imaging phantom, *in vivo* estimation of ferumoxytol or exogenous iron by QSM is much more complex due to tissue heterogeneity and uneven distribution of susceptibility sources. To overcome these challenges, we applied the graph-cuts based simultaneous phase unwrapping and chemical shift removal method (SPURS)^33^ to correct phase and chemical shift discontinuities in the MRI gradient-echo data (Fig. 2a). QSM was then reconstructed from the unwrapped field map with the preconditioned total field inversion algorithm^34^. The steps for deriving QSM in this study differed from our prior studies^19^ because of the need to correct for a significant chemical shift from the high fat present in mouse torso and the need to estimate ferumoxytol in a range of concentrations suitable for MFH applications. With this advanced QSM technique, the area injected with ferumoxytol in live mice was clearly discernible from the neighboring tissues (a yellow circled region in Fig. 2a). However, the high susceptibility region was not entirely confined to the injection site; one should therefore rely on the location of SPIO injection site or perform MR scans prior to and post SPIO injection to differentiate the specific susceptibility of SPIO from non-specific susceptibility noise. The difficulty with QSM for quantifying SPIO *in vivo* is also apparent in the coronal view (Fig. 2b). With algorithms for reducing most of the streaking artifacts, intravenously injected ferumoxytol distribution in the liver could be clearly distinguished from other susceptibility sources. In the same mouse that was implanted with tumor by subcutaneous injection of tumor cells into the upper flank, ferumoxytol accumulation in the tumor was also discernible, displaying higher susceptibility at the periphery of the tumor (yellow circled region in Fig. 2b). The distribution of nanoparticles such as ferumoxytol at the boundary between the tumor and neighboring tissues seemed to be consistent with prior observations, which was ascribed to a phenomenon referred to as the enhanced permeability and retention (EPR) effect^35–37^.

**Figure 3:**
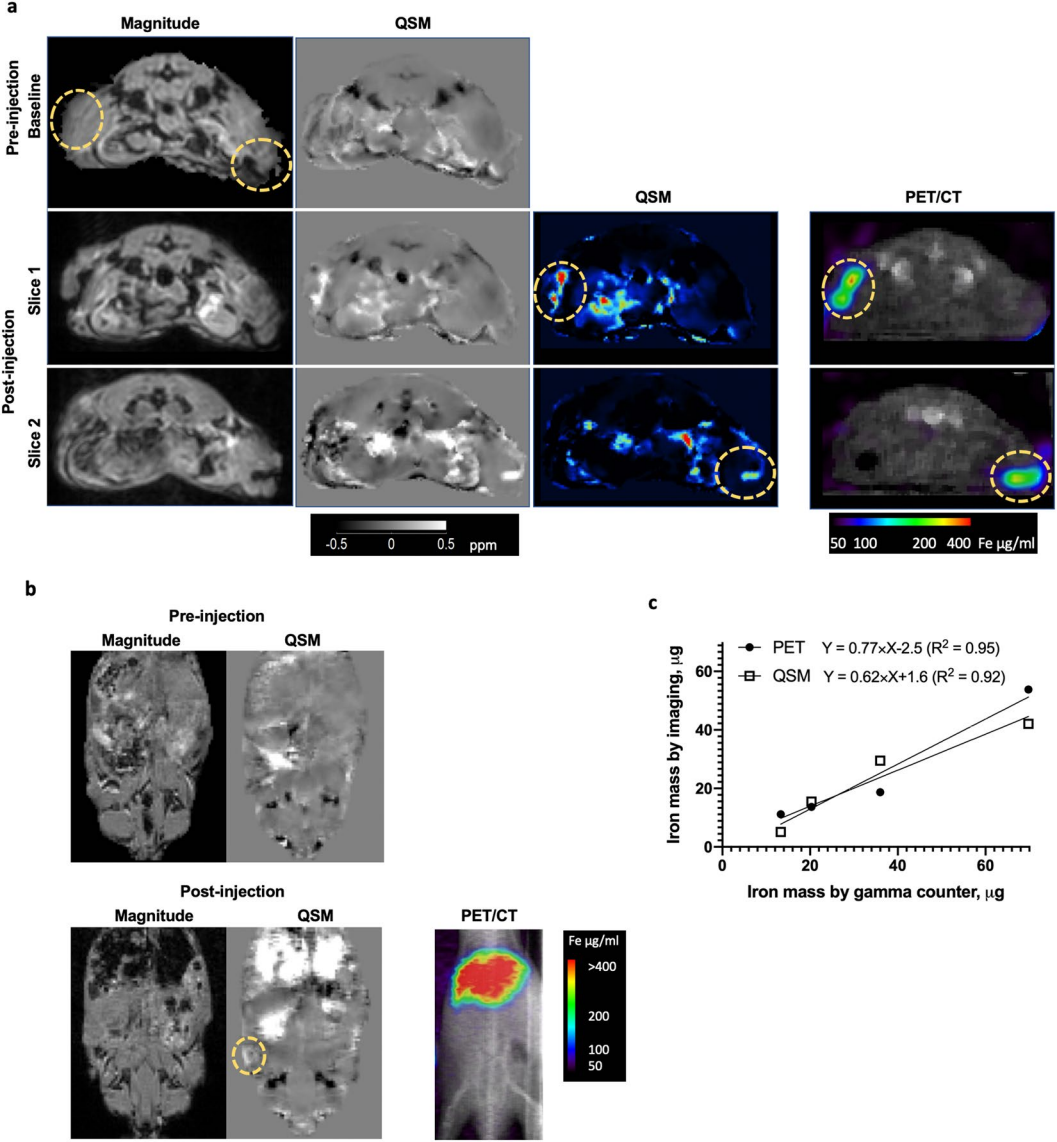
Validation of MR QSM by comparison with PET and gamma counter measurement. (**a**) MRI (magnitude and QSM) images obtained before and after local injection of ^89^Zr-ferumoxytol. PET/CT images are shown after injection of ^89^Zr-ferumoxytol. The yellow circles on the baseline images for MRI show the location of the implanted tumors. Two slices of the MRI and PET images acquired after intratumoral injection of ^89^Zr-ferumoxytol are shown in the next two rows. (**b**) QSM and magnitude images of mice with subcutaneous tumor imaged before and 24 hours after tail-vein injection with ^89^Zr-ferumoxytol (3 mg Fe). The yellow circle denotes QSM detection of ferumoxytol distribution in tumor lesion. PET images are shown for comparison. (**c**) Imaging-based estimation of intratumorally injected ferumoxytol in live subjects was validated by comparison with the gamma counter measurement of ferumoxytol in harvested tumors.

With the QSM algorithm fully optimized for *in vivo* detection of SPIO, we then examined the accuracy of QSM using ^89^Zr-ferumoxytol by comparing QSM with PET estimates (Fig. 3a). Mice were scanned pre- and post-ferumoxytol injection to discern susceptibility changes due to SPIO injection. To minimize the dissipation of ferumoxytol from the injection site to outside of tumors and to test the accuracy of QSM at a lower range, we chose the injection volume of ferumoxytol to be 1% of the tumor volumes. The injection sites at tumors near the flank were clearly visible with QSM intensities. We also noted close resemblance of the peak intensities delineated by QSM and PET in both sides of tumors. After PET/CT and MRI scans were completed, mice were sacrificed and subcutaneous tumors excised for gamma counter measurement to validate imaging-based estimation of ferumoxytol in live subjects. The amount of ferumoxytol predicted by PET and QSM corresponded linearly to the estimate by gamma counter except for PET and QSM’ underestimation of ferumoxytol (slope 0.77 and 0.62 for PET and QSM versus gamma counter, respectively, shown in Fig. 3c and Table 1). The percent of ferumoxytol retained within the tumors, determined by the difference between the actual injection amount and gamma counter measurement, was essentially 100% for 2 out of 4 tumors, while it was only about 50% for the other 2 tumors. The loss of retention was likely due to dissipation of ferumoxytol to outside of tumors or inaccuracies with delivering microliter volumes into tumors.

**Table 1:** Comparison of ferumoxytol accumulation measured by QSM, PET, and gamma counter.

Tissue	Volume^4^ (mm^3^)	Mass of Iron (μg)			Amount injected	
		Gamma counter	PET	QSM	Mass, μg	Volume, μl
Tumor^1^	345	69.8	53.8	42.1	69.1	3.5
	221	20.3	13.7	15.5	44.3	2.2
	182	36.0	18.7	29.5	36.3	1.8
	147	13.3	11.2	5.1	29.5	1.5
Liver^2^	1,975	336	352.3	354.0	3,000	150
	1,890	349	374.4	*n.d*.	3,000	150
	1,981	322	354.0	*n.d*.	3,000	150
Tumor^3^	213 ± 69	*n.d*.	2.0 ± 0.5	1.4 ± 0.4	3,000	150

1. Ferumoxytol (20 mg/ml) was injected intratumorally with the volume equal to approximately 1% of the tumor volume. Immediately after injection, live mice were scanned by PET and MRI for QSM. Gamma counter was performed on excised tumors.

2. Ferumoxytol (20 mg/ml, 150 μl) was injected intravenously via tail vein. At 24-h post-injection, live mice were scanned by PET and MRI for QSM. Gamma counter was performed on the harvested liver. *n.d*. = not determined.

3. Ferumoxytol (20 mg/ml, 150 μl) was injected intravenously via tail vein to mice with subcutaneous tumors (n = 4). At 24-h post-injection, live mice were scanned by PET and MRI for QSM.

4. The volume of tumor and liver was estimated by VOI defined based on MRI and CT images.

In comparison with subcutaneous injection, intravenous injection of ferumoxytol into mice with subcutaneous tumors resulted in uptake mainly by the liver (Fig. 3b). At 24 h post tail-vein injection of ferumoxytol, less than 0.1% of injected dose was found in tumor sites, while 11% of injected dose was accumulated in the liver (Table 1). The uptake of ferumoxytol by the liver (~185 μg/ml) in mice was comparable to the levels reported for human subjects (~140 μg/ml)^38^. In comparison, the amount of ferumoxytol in our subcutaneous PC3 tumors (~10 μg/ml) was significantly lower than the levels in tumor lesion in the same study (median value ~ 34.5 μg/ml)^38^. Dominant liver uptake of ferumoxytol is consistent with the clinical use of ferumoxytol (that is digested by macrophages in the liver to release iron ions to the blood) to treat iron deficiency anemia in chronic kidney disease patients^39^. Likewise, after imaging of live subjects by PET/CT and MRI for QSM, the liver was harvested and processed for gamma counter. The QSM and PET estimates of ferumoxytol accumulation in the liver were found to be in excellent agreement (<6% deviation) with the gamma counter estimate (Table 1).

**Figure 4:**
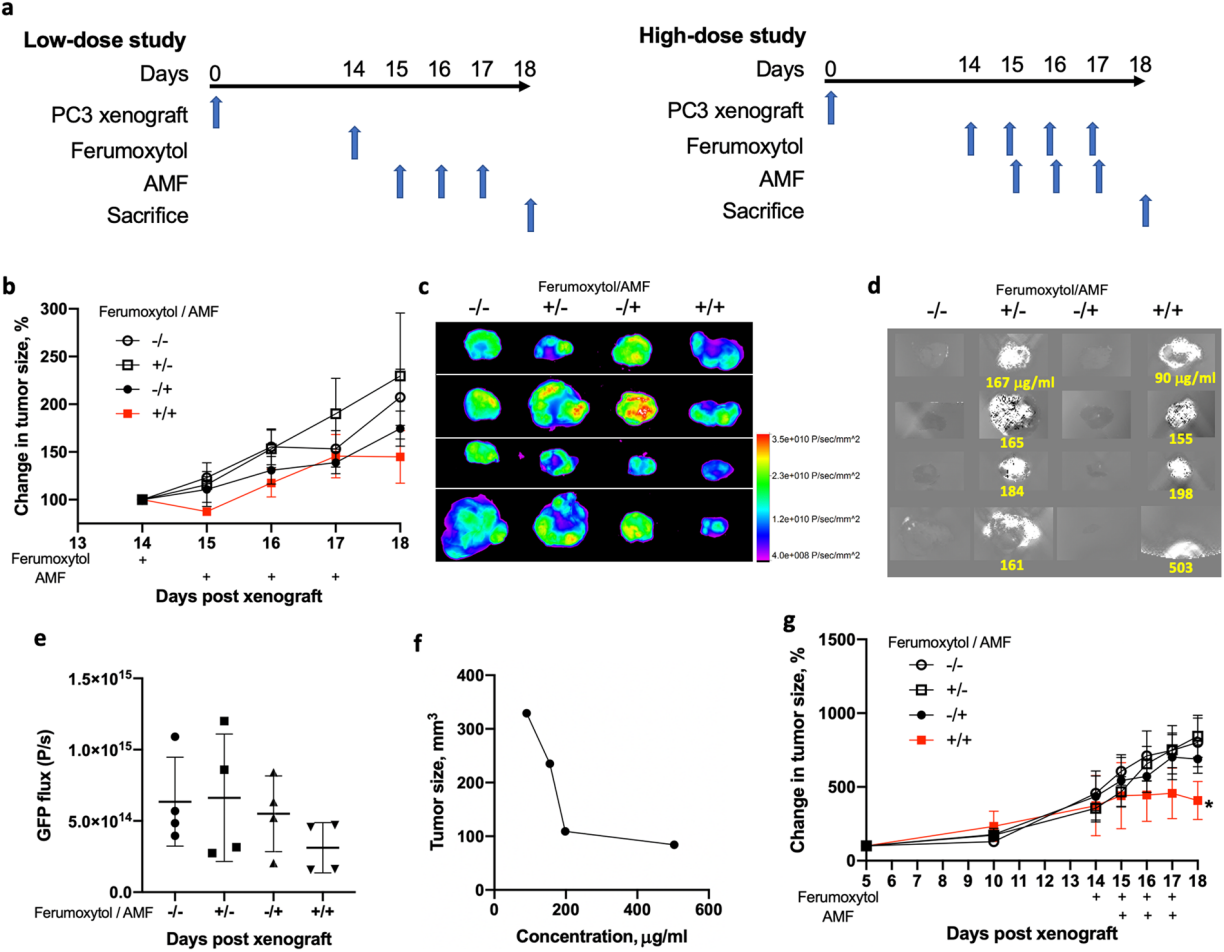
Ferumoxytol and hyperthermia treatment on subcutaneous PC3 xenografts. (**a**) Schematics of single, low-dose and multiple, high-dose study design are shown. Single, low-dose ferumoxytol study (**b–f**): (**b**) mice were assigned to four cohorts (−/−, +/−, −/+, +/+ of ferumoxytol/AMF) for the combination of ferumoxytol (5 mg/ml, 25 μl) and AMF treatments (n = 4). Tumor size was measured daily during ferumoxytol/AMF treatments. (**c**) PC3 tumors were harvested on day 18 (24 h after the last AMF application), fixed, and imaged for GFP fluorescence to estimate PC3 tumor cell viability. (**d**) Immediately after *ex vivo* imaging of tumors by fluorescence, the same tumors were imaged by MR (parameters used were Multi-echo Gradient Echo, TE1/ΔTE: 3.9 ms/ 4.8 ms, voxel size: 0.2433 mm^3^, TR: 22.5 ms, flip angle: 150°) to estimate ferumoxytol amounts. Numbers in yellow indicate average ferumoxytol concentrations (μg/ml) within tumors. (**e**) Quantification of total GFP flux is shown for the tumors in the four different cohorts of the single, lose-dose ferumoxytol study. (**f**) Tumor size versus average ferumoxytol concentrations within tumor is plotted. Multiple, high-dose ferumoxytol study (**g**): mice were assigned to four cohorts (−/−, +/−, −/+, +/+ of ferumoxytol/AMF) for the combination of ferumoxytol (four cycles of 30 mg/ml at 25 μl) and AMF treatments (3 cycles; n = 4). By paired t-test for the measurements on days 16–18, *p < 0.05 for +/+ vs other controls.

We then examined the therapeutic efficacy of ferumoxytol at two different doses and AMF-induced hyperthermia on suppression of subcutaneous tumor growth (Fig. 4a). Immuno-deficient NSG mice were utilized as a host to accommodate the growth of human cell line, and to avoid possible hyperthermia-induced activation of immune cells and their effects on tumor killing. When tumors of PC3 prostate cell line reached approximately 100 mm^3^ in size, mice were grouped into no treatment control or three treatment cohorts that were subjected to intratumoral injection of ferumoxytol, AMF, or both (Fig. 4b). To examine the effect of hyperthermia induced cell killing in its relation to ferumoxytol distribution, we chose to use a small dose of ferumoxytol (a single injection of 25 μl at 5 mg/ml, *i.e*., 125 μg) to avoid bulk heating-induced bystander killing. The combination of ferumoxytol and AMF led to measurable inhibition of tumor growth compared to no treatment or either the ferumoxytol- or AMF-only treatment cohorts. However, the single injection, low-dose ferumoxytol plus AMF combination was only marginally significant at reducing tumor size (p = 0.06 for +/+ vs. −/−) (Fig. 4b). At 24 h after the last treatment, mice were sacrificed and tumors were taken out for *ex vivo* fluorescence imaging of GFP intensity to assess tumor viability (Fig. 4c,e). Following fluorescence imaging, excised tumors were subsequently scanned by MRI for QSM to relate tumor killing to ferumoxytol concentration (Fig. 4d). The amount of ferumoxytol retained within the tumor was lower overall than the amount injected (125 μg), likely caused by dissipation of ferumoxytol during the four days after intratumoral injection, ferumoxytol degradation inside cells, and/or the loss of SPIO properties due to exposure to AMF. However, judging from a trend of lower tumor volume with increasing ferumoxytol concentrations within tumors (Fig. 4f), hyperthermia effects was indeed responsible for tumor killing even at this low dose of ferumoxytol injections. To further examine if tumor killing correlates with ferumoxytol dose, a repeated, high concentration of ferumoxytol (four injections of 25 μl at 30 mg/ml, *i.e*., 750 μg per injection) was used. As anticipated, the combination of high dose ferumoxytol and AMF led to significant reduction in tumor size (Fig. 4e).

**Figure 5:**
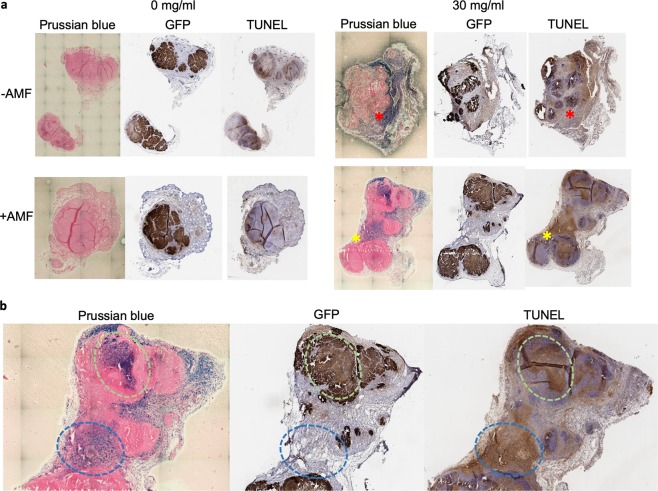
Histological examination to confirm hyperthermia-induced tumor killing. (**a**) Adjacent tumor sections were stained for Prussian blue to detect iron, GFP to detect viable tumor cells, and TUNEL to detect apoptotic cells. Tumor slices were obtained from four cohorts of ferumoxytol/AMF combinations. Red asterisk is placed to a region of Prussian blue positive and TUNEL negative regions. Yellow asterisk is placed to a region of Prussian blue positive and TUNEL positive. (**b**) Zoom-in view of the slice of tumor from +/+ of ferumoxytol/AMF cohort. Green circle indicates the site of intratumorally injected ferumoxytol. Blue circle is placed on tumor stroma (GFP negative) that stained positive both for Prussian blue and TUNEL.

The histology of subcutaneous tumors receiving intratumoral injections of ferumoxytol with or without AMF further confirmed the specific killing induced by MFH (Fig. 5). First, we noted that even with intratumoral injection, ferumoxytol (delineated by Prussian blue stain) appeared to distribute to the periphery of tumors rather than permeating evenly throughout tumor tissues. Due to a small volume of intratumoral injection of ferumoxytol, we noted that hyperthermia induced tumor killing (identified by TUNEL stain) appeared to be restricted to the region of ferumoxytol localization, validating a lack of temperature elevation in the bulk of the tumor. Cell death in the tumor stroma (a region marked with yellow asterisk in Fig. 5a and blue dotted circle in Fig. 5b) was also confirmed, identified by the absence of colocalized GFP staining with ferumoxytol distribution. Co-localization of ferumoxytol distribution and cell death by TUNEL was less apparent in tumors without AMF (a region marked with red asterisk; Fig. 5a).

## Discussion

Magnetic susceptibility, a fundamental physical property of a SPIO contrast agent such as ferumoxytol is proportional to the concentration of contrast agent and independent of its surrounding medium. The molar susceptibility of the contrast agent, which can be predetermined using phantom calibration *in vitro*, can be used to convert a susceptibility map into a concentration distribution of SPIO contrast agent *in vivo*. Quantitative imaging of SPIO distribution *in vivo* is not only useful as a blood pool contrast agent but can potentially aid magnetic hyperthermia in cancer by providing a better prediction of temperature rise to ensure therapeutic activity in tumor tissues while minimizing unwanted side effects^40^. Previously, we reported the application of QSM to estimate SPIO distribution in animals after fixation, and validated the accuracy of QSM on harvested tissues^28,29^. In this study, we applied QSM technique to estimate and validate distribution of clinically approved SPIO nanoparticles in live subjects, which will be necessary for predicting temperature elevation in tissues in response to AMF *in vivo*.

QSM prediction of SPIO directly injected into solid tumors near the skin faces technical challenges due to the need to correct for a chemical shift arising from the high fat content near the skin, the proximity of tumor growth to the skin in contact with air, and a large variation of SPIO concentrations near the injection site. Tissue susceptibility due to high fat in the skin was eliminated by the SPURS method in this study^33^, which was specifically designed to correct for chemical shift contributions to the phase in the gradient-echo MRI data. SPURS has been shown to produce more accurate QSM maps as it avoids the need for additional field map smoothing^33^. Due to the proximity of tumor growth near the skin and air interface, we used QSM reconstruction that avoids the removal of the background field as this technique assumes that the susceptibility source is far from the tissue edge and its field is approximately orthogonal to the background field^18,34^. Another difficulty encountered in this study was the degree of susceptibility source variation that ranged from ~0.1 ppm for soft tissue to high ppm values (e.g., 348 ppm for 30 mg/ml SPIO) for intratumoral injections of SPIO. We used the recently reported preconditioned total field inversion QSM technique^34^ to overcome these problems. The resulting QSM algorithms developed in this study enabled the mapping of SPIO distribution in the entire body of live subjects *in vivo*. From the QSM map, intravenously injected ferumoxytol in mice with subcutaneous tumor xenografts was found mainly in the liver. Ferumoxytol accumulation in the tumor was clearly discernable, but predominated at the tumor-stroma boundary. This finding is consistent with many prior studies demonstrating accumulation of nanoparticles and antibodies around the tumor periphery^41,42^. Our QSM therefore can be used for evaluating biodistribution and pharmacokinetics of SPIO nanoparticles in real-time and for exploring different designs of nanoparticles with improved tumor targeting.

SPIO nanoparticles are being investigated as therapeutic agents for hyperthermia-induced tissue heating under AMF^1,3,40^. Inhomogeneous distribution of SPIO after intratumoral injection has previously been attributed to uneven tumor growth inhibition^3^. Therefore, *in vivo* quantification of SPIO such as ferumoxytol by a clinical imaging modality can provide information for predicting temperature elevation under AMF in live subjects, and therefore insight into possible cellular and tissue damage. To correlate SPIO concentration with tumor killing by hyperthermia, two different doses of ferumoxytol were used for intratumoral injection. For a low-dose study, ferumoxytol was injected 24 h prior to AMF application, which was chosen to promote cellular internalization of ferumoxytol. For the high-dose, repeated ferumoxytol study, the first ferumoxytol injection was made 24 h prior to AMF, followed by three cycles of ferumoxytol injection and immediate application of AMF. The dependence of cell killing on ferumoxytol concentration and AMF application was apparent, judging from the trend of tumor size and ferumoxytol amounts within tumor, and more significant tumor killing effects seen in the high-dose cohort. Compared to more pronounced suppression of tumor growth in prior studies^1,3,43^, MFH effects seen in this study were more modest. This was likely due to a relatively low specific absorption rate of ferumoxytol compared to other SPIOs^31^, a delivery of small injection volume into the tumor, and the use of immune-compromised host that lacks in a hyperthermia-induced immune or macrophage-mediated killing of tumors^44,45^. It is also likely that further optimization including ferumoxytol dose, route of injection, timing of injection and AMF application, and AMF parameters will significantly influence the efficiency of tumor killing. Hyperthermia-induced tumor killing will be critically influenced by tumor and stroma interactions and the degree of tumor blood vessel formation, which will affect EPR effect for SPIO uptake by tumor both for intratumoral and intravenous routes of injection. The goal of our current study, however, was not achieving maximum MFH-induced tumor killing, but rather developing and demonstrating a clinical imaging tool to quantitatively assess SPIO distribution in live subjects, and correlating its distribution with cell killing directly caused by MFH.

Compared with PET imaging of radionuclides bound to iron-oxide nanoparticles for iron quantification, our QSM approach is advantageous in that it avoids the use of ionizing radiation and provides superior soft tissue contrast and spatial resolution. QSM can serve multiple purposes to improve MFH by mapping SPIO distribution prior to AMF. In particular, it can be used to optimize intratumoral delivery of SPIOs to ensure SPIO dispersion throughout the tumor while minimizing distribution into normal tissues for more accurately targeted hyperthermia. More broadly, the QSM algorithms developed in this study can facilitate preclinical and clinical studies to explore and optimize SPIO-based hyperthermia therapy in terms of material design, SPIO and AMF dose and frequency, as well as the route of SPIO delivery.

## Methods

### Preparation of dual-modality contrast agent ^89^Zr-ferumoxytol

^89^Zr-conjugated ferumoxytol (AMAG Pharmaceuticals) was produced using 1-Ethyl-3-(3-dimethylaminopropyl) carbodiimide (Sigma) and DFO-p-SCN (Macrocyclics) following a procedure described previously^9^. For validation of QSM by PET, imaging phantom containing ^89^Zr-ferumoxytol ranging from 1.8 to 117.0 μg/ml by mass of iron (Fe) was prepared by serial dilution of ^89^Zr-ferumoxytol suspended in 1% agarose in PBS solution successively layered on beds of 1% agarose gel in a falcon tube.

### Animal experiments

All animal experiments were performed in strict accordance with the recommendations contained within the National Institute of Health’s Guide for the Care and Use of Laboratory Animals. Animal handling protocols were approved by the Institutional Laboratory Animal Use and Care Committee of Weill Cornell Medicine (Permit Number: 2012–0063). Four to six week old non-obese diabetic/Cg-*Prkdc*^*scid*^ *Il2rg*^*tm1Wjl*^/SzJ (NSG) mice (Jackson Laboratory) were injected subcutaneously in the flank with 5 × 10^6^ PC3 (prostate cancer cell, ATCC) cells in 1:1 mixture of PBS, pH 7.4 and Matrigel (BD Biosciences, Bedford MA). PC3 cells were stably transduced with GFP-encoding lentiviral vector (Biosettia) to locate tumor cells by immunostaining and viability by fluorescence. Tumor growth was monitored by taking caliper measurements of the three orthogonal dimensions of the tumor and approximating its volume using an ellipsoid equation.

### *In vivo* validation of QSM by PET and gamma counter

For validation of MR QSM in live subjects, ^89^Zr-ferumoxytol was injected directly into tumors when tumor size reached approximately 100 to 250 mm^3^. The injection volume of ^89^Zr-ferumoxytol (20 mg/ml) was set to 1% of the tumor volumes, which was chosen to test the lower limit of the imaging sensitivity and to minimize the loss of ferumoxytol due to dissipation from tumors. Live mice were scanned by PET/CT and MRI prior to and immediately after injection of ferumoxytol. After imaging, mice were euthanized, and subcutaneous tumors were harvested and processed for the measurement of weight and gamma count to obtain absolute amount of ^89^Zr-ferumoxytol retained in the tumor. In a second group, ^89^Zr-ferumoxytol (20 mg/ml, 150 μl) was injected intravenously via tail vein. At 24 h post-injection, mice were scanned by PET/CT and MRI for QSM. After imaging, mice were euthanized and the liver was harvested for the measurement of weight and gamma count.

### Hyperthermia

Magnetic fluid used in this study is ferumoxytol, which is SPIO coated with polyglucose sorbitol carboxymethylether^46^. The overall colloidal particle size is 17–31 nm in diameter^47^. Using a magnetic hyperthermia system (MSI Automation) with a frequency of 335 kHz and magnetic field strength of 60 kA/m, a rise in temperature of ferumoxytol at 1 mg/ml was 0.985 C°/min with specific absorption rate of 68.7 W/g, close to the values reported previously^46^. At 14 days post tumor xenograft (~100 mm^3^ in size), mice were assigned to four cohorts for hyperthermia experiments, which were performed at two different doses of ferumoxytol. For single, low-dose ferumoxytol study, mice were injected intratumorally with 25 μl of 5 mg/ml ferumoxytol and at 24 h post injection subjected to AMF. For multiple, high-dose ferumoxytol study, mice were first injected intratumorally with 25 μl of 30 mg/ml ferumoxytol 24 h prior to the three cycles of ferumoxytol (25 μl of 30 mg/ml) and AMF applied immediately after injection. Injections were made at 3 μl/min with an automatic syringe pump (Harvard Apparatus). Mice were anesthetized by ketamine/xylazine cocktail intraperitoneal injection and were subjected to AMF (335 kHz and 14.5 kA/m) for 45 minutes. At 24 h after the final application of AMF, mice were anesthetized with an intraperitoneal injection of a ketamine/xylazine combination (150 mg/kg and 15 mg/kg, respectively) and underwent cardiac perfusion with PBS followed by 4% paraformaldehyde to flush out blood within tissues. Subcutaneous tumors were harvested, weighed, and fixed for 24 hours in 4% paraformaldehyde, and imaged for fluorescence (*In-Vivo* F-Pro, Bruker) to assess tumor viability.

### PET/CT and MR imaging of mice

PET (Siemens Inveon PET/CT scanner) imaging of the mice was performed using list mode acquisition for 30 minutes with a gamma-ray energy window of 350 to 650 keV, a coincidence timing window of 3.4 ns, and OSEM3D/MAP reconstruction. Immediately after PET/CT scans, MRI was performed with a 72 mm diameter, linear, transmit/receive coil (7 T Bruker Biospec USR 70/30 preclinical MRI scanner). Mice were kept under 2.5% isoflurane-induced anesthesia during imaging. MRI data were acquired using a three-dimensional multi-echo gradient echo (GRE) sequence with the following parameters: voxel size = 0.4 mm isotropic, field-of-view = 35 × 35 mm^2^, bandwidth = 763 Hz/pixel, flip angle = 15°, echo time (TE1/ΔTE) = 3 ms/2.3 ms, repetition time (TR) = 22.1 ms, number of echoes = 12, signal averages = 6.

### Image reconstruction and post-processing

QSM maps were computed offline from multi-echo GRE MRI data using custom MATLAB software (version R2012b, The Mathworks, USA). The susceptibility induced field inhomogeneity map, *f*, was estimated by a nonlinear voxel-wise fit to the phase of the complex data, unwrapped and corrected for chemical shift using the graph-cut based SPURS algorithm^25,33,48^. A binary mask, *M*, isolating the region of the anatomy with the tumor, was created by applying a region-growing algorithm to the MRI magnitude image in the ITK-Snap image processing software^49^. The susceptibility map was then estimated by solving the equation for preconditioned total field dipole inversion:

$${\chi }^{\ast }=P{y}^{\ast }\,{\rm{s}}{\rm{.t}}.{y}^{\ast }=arg\,mi{n}_{y}\frac{1}{2}||w(f-d\ast Py)|{|}_{2}^{2}+\lambda ||{M}_{G}\nabla Py)|{|}_{1}$$

where *χ* is the total susceptibility, * is its convolution with the dipole kernel *d*, *f* is the total field, *w* is a noise weighting, ∇ is the gradient operator, and *M*_*G*_ is the binary edge weight for suppressing streaking artifacts. The values in the preconditioner matrix, *P*, which accounts for strong susceptibility differences between the high SPIO dose and surrounding tissue in the tumor, were set to 30 for air (region outside the mask) and to the signal to noise ratio matrix for tissue (region inside the mask) estimated from the phase fitting step^34^. The optimal regularization parameter, *λ*, was chosen empirically and set to 1000. MRI and PET/CT images were manually aligned using the AMIDE multimodality image analysis software (AMIDE software; http://amide.sourceforge.net/index.html). Volumes of interest (VOIs) with dimensions equal to the tumor volume were drawn on the PET/CT and MRI images to cover the visible tumor volume. The mean voxel values and volumes from the VOIs were recorded for each image type. Using a fat mask created by binarization of the fat map from the fat/water separation procedure, the average QSM of fat in the mouse was estimated, used as reference for VOI estimates. All mean voxel values were converted into total iron mass using the coefficients determined from the phantom calibration experiments.

### Gamma count

Organs were harvested from euthanized specimens and scanned individually using Wizard 2 Gamma Counter (Perkin Elmer). Harvested tumors and liver were weighed, and a fraction of these tissues (1/4-1/2 of the total volume) was used for gamma counter. Organs were not washed in order to maximize the retention of ^89^Zr-ferumoxytol. A standard of ^89^Zr-ferumoxytol was prepared to calculate absolute iron uptake in each organ.

### Histology

Mice were anesthetized with an intraperitoneal injection of a ketamine/xylazine combination (150 mg/kg; 15 mg/kg) and underwent cardiac perfusion with PBS and subsequently 4% paraformaldehyde by a trained animal user according to IACUC guidelines. Subcutaneous tumors were fixed for 24 hours and embedded in paraffin and cut in 7 µm sections (Microtome, Leica). Sections were stained for iron accumulation by Prussian Blue staining^29^, to detect DNA breaks by TUNEL (terminal deoxynucleotidyl transferase dUTP nick end labeling) stain, and to localize tumor growth by GFP immunostaining (performed by Histowiz, Inc.).

## Data Availability

All data generated or analyzed during this study are included in the published article.
